# Editorial: Molecular determinants of protein assemblies in health and disease

**DOI:** 10.3389/fmolb.2022.1107686

**Published:** 2022-12-14

**Authors:** Sonja Kroschwald, Anoop Arunagiri, Salvador Ventura, Srivastav Ranganathan, Verena Kohler

**Affiliations:** ^1^ Institut für Biochemie, ETH Zurich, Zurich, Switzerland; ^2^ Department of Internal Medicine, University of Michigan, Ann Arbor, MI, United States; ^3^ Institut de Biotecnologia i Biomedicina and Departament de Bioquímica i Biologia Molecular, Universitat Autònoma de Barcelona, Bellaterra, Barcelona, Spain; ^4^ Department of Chemistry and Chemical Biology, Harvard University, Cambridge, MA, United States; ^5^ Institute of Molecular Biosciences, University of Graz, Graz, Styria, Austria

**Keywords:** protein structure-function relationship, protein aggregation, amyloid, biomolecular condensates, molecular chaperone, neurodegenerative diseasaes, gut microbiome, bacterial amyloid

Protein structure determines its biological function. Conceptually, protein structure can be classified as folded or “natively-unfolded”. While the defined fold of the former dictates a unique function, the latter also called “intrinsically-disordered”, allows polypeptides to engage in a multitude of interactions. Structural features determine also the self-assembly propensity of a protein. Beyond the intrinsic structural aspect, a protein’s tendency to form higher-order assemblies can be influenced by interacting proteinaceous/non-proteinaceous biomolecules. The material properties of assemblies that are not membrane-enclosed range from liquid to gel-like biomolecular condensates to solid-like aggregates. These assemblies can serve to fulfill various biological functions, including fostering a reaction, for instance, increasing the activity of actin regulatory proteins [*Science 2019; 363(6431):1093-97*], or protecting proteins, e.g. preventing stress-induced damage of the translation termination factor Sup35 [*Science 2018; 359(6371):eaao5654, reviewed in Nature Reviews 2021; 22: 196–213*]. Correspondingly, they play fundamental roles at cellular and organismal levels. However, if the regulatory mechanisms of these assemblies are error-prone, aggregation-related diseases ensue. These ideas inform us that 1) protein structure, 2) nearby physical interactors, and 3) cellular regulatory elements overall serve as the molecular determinants of protein assemblies. The contributing articles in this Research Topic explore protein structure-function, structure-aggregation, and aggregation-disease relationships.

“Exploring the eukaryotic Yip and REEP/Yop superfamily of membrane-shaping adapter proteins (MSAPs): A cacophony or harmony of structure and function?”, by (Angelotti) is a comprehensive review that explores the Yip (Ypt-interacting protein) family proteins, their binding partners, and cellular functions that mostly encompass membrane shaping. The article epitomizes classic examples of dealing with and debating topology-to-function links of proteins.

As introduced, proteins have evolved to self-assemble and acquire new or additional functions. An interesting class of solid-like aggregates is amyloids, characterized by a beta-sheet-rich fibrillar structure. Amyloids have been shown to have functional roles in all kingdoms of life. They are associated with storage in eukaryotes or with biofilm formation to confer antibiotic resistance and host colonization in fungi. Involved in the latter is adhesin Als5 from *Candida* albicans, which structural features are addressed in the article by Golan et al. “Structure and conservation of amyloid spines from the *Candida* albicans Als5 adhesin” in this Frontiers collection.

Various mechanisms monitor and regulate the formation, properties, dynamics, and dissolution of biomolecular condensates and aggregates/amyloids, such as posttranslational modifications, and the protein quality control system, consisting of molecular chaperones and the protein degradation machinery. The article “The Impact of Hidden Structure on Aggregate Disassembly by Molecular Chaperones” by Shoup et al. addresses the role of the bi-chaperone (Hsp70/Hsp100) disaggregase system from E. coli on aggregates with a particular focus on aggregate internal structure. The authors show that aggregates form particles resistant to chaperone-mediated disassembly over time. This transitioning of assemblies into more solid-like states has been observed for a number of phase-separating and aggregating proteins and can be associated with a variety of age-related diseases. For example, the nucleating core of the Alzheimer’s disease-related amyloid-beta 42 peptide assembles through a liquid-like state into the characteristic amyloid cross-ß structure. The study by Gordon-Kim et al. about “Polyanion order controls liquid-to-solid phase transition in peptide/nucleic acid co-assembly” demonstrates how nucleic acid structures can externally template this liquid-to-solid transition. The authors propose mechanisms for templating events in condensation and amyloid nucleation/propagation events that might be applicable to other assemblies.

Huntington’s disease is another well-characterized neurodegenerative protein misfolding disorder, caused by pathogenic polyQ expansion in the N-terminal huntingtin (HTT) protein that drives the protein to misfold and aggregate into amyloid fibrils. Many protein misfolding diseases are associated with the occurrence of missense mutations. The paper from Dinamarca et al. “Synaptic and functional alterations in the development of mutant huntingtin expressing hiPSC-derived neurons” illustrates how hereditary disease mutations influence early development processes of the individual and the way this might impact the progression of diseases thought to be age-related.

Parkinson’s disease, yet another widely studied neurodegenerative disorder, is characterized by the intracellular deposition of amyloid fibrils in the brain tissues. It is proposed that the prefibrillar oligomeric states and not the mature amyloid fibrils contribute primarily to disease progression, thus being the cytotoxic species. Yoo et al. discuss in “Polymorphism in alpha-synuclein oligomers and its implications in toxicity under disease conditions” how the different shapes of oligomers could differentially contribute to neurotoxicity.

In addition to genetic and internal regulatory mechanisms, several extrinsic factors might modulate the formation of amyloid states and, ultimately, cross-seeding events. The review “Microbiome Impact on Amyloidogenesis” by authors Curto et al. summarizes the evidence about how amyloid structures formed by microorganisms could cross-seed the amyloid conformations of human proteins and contribute to the onset of diseases. They also highlight the manipulation of the microbiome as a therapeutic strategy.

Biomolecular condensates are further gaining center stage as potential drug targets. Many condensate-forming proteins are enriched in intrinsically disordered regions (IDRs) which are often necessary to build up the network of sustaining condensates. However, their unique properties make them also prone to aggregate under non-physiologically settings. Identifying condensates as drug targets holds the potential of impacting these otherwise undruggable IDRs. The current efforts are summarized by Patel et al. in “Principles and functions of condensate modifying drugs” where they showcase different categories of condensate modifiers or c-mods.

Overall, in this issue, we present a bouquet of novel research plus timely review articles highlighting protein structure-function connection (Angelotti) and self-assembly including the functional role of solid-like assemblies (Golan et al.). Other studies here concentrate on the factors including microbiota affecting amyloidogenesis (Curto et al.), developmental outcomes of aggregation-prone protein mutations (Dinamarca et al.), proteotoxicity exerted by oligomers (Yoo et al.), and naturally-occurring aggregation modulators like nucleic acid (Gordon-Kim et al.) or chaperones (Shoup et al.), which help tune the state of these assemblies. These key molecular insights along with a report on potential alleviating strategies against aggregation disorders (Patel et al.) make this a well-rounded compilation of complementary and unique articles. We believe this topic would be of interest to biophysicists, evolutionary biologists, material scientists, and cell biologists alike.

